# Supplementary material for: Effects of supplemental oxygen on systemic and cerebral hemodynamics in experimental hypovolemia: Protocol for a randomized, double blinded crossover study
Source: PLoS One. 2022 Jun 24;17(6):e0270598. doi: 10.1371/journal.pone.0270598 (PMC9231698; doi:10.1371/journal.pone.0270598)
Supplement: S1 Protocol — (PDF) [file pone.0270598.s002.pdf]

[redacted]

Effects of supplemental oxygen on systemic and cerebral hemodynamics in experimental hypovolemia

A randomized, phase I, crossover study to study the effect of supplemental oxygen vs. room air on cerebral and systemic hemodynamics in healthy volunteers > 18 years during experimental hypovolemia in the lower body negative pressure model of hypovolemia

Protocol Number: 4_14-12-2021

Amendment Number:

Compounds: V03A N01 Oxygen

Brief Title: Supplemental oxygen in hypovolemia

Study Phase: I

Sponsor Name:

Oslo University Hospital

Anne Bøen, Acting Head of Department.

Department of Anesthesiology

Division of Emergencies and Critical Care

Oslo University Hospital

P.O.Box 4956, Nydalen

0424 Oslo

Manufacturer: Linde, AGA Gas AB, Sverige, Linde Healthcare AB, Sverige, Linde Gas, Sverige; Repr. Linde, Linde Gas AS (tidligere AGA), Gjerdrumsvei 8, 0484 Oslo

Regulatory Agency Identifier Number(s)

EudraCT: 2021-003238-35

ClinicalTrials.gov: NCT05150418

REK (Regional Ethics Committee): 285164

PVO (Data Protection Officer): 21/19016

Approval Date:

Statens legemiddelverk: 19-08-2021

REK (Regional Ethics Committee): 15-09-2021 (approved with conditions)

[redacted]

Sponsor Signatory:

[redacted]

Anne Bøen MD **Date**

Head of Department

Medical Monitor Name and Contact Information:

Fridtjof Heyerdahl MD, PhD
Department of Research,
Norwegian Air Ambulance Foundation,
Postboks 414 Sentrum,
0103 Oslo
Norway

[redacted]

Protocol Amendment Summary of Changes Table

DOCUMENT HISTORY	
**Document**	**Date**
Original Protocol	*08-06-2021*
Protocol_2_06-08-2021	*06-08-2021*
Protocol_3_01-10-2021	*01-10-2021*
Protocol_4_14-12-2021	*14-12-2021*

[redacted]

Contact Details

**Sponsor:**	**Name and title:**<br>**Anne Bøen, MD, acting head of department**<br>Address:<br>Department of Anesthesiology<br>Division of Emergencies and Critical Care<br>Oslo University Hospital<br>P.O.Box 4956, Nydalen<br>0424 Oslo<br>[redacted]
**Principal Investigator:**	**Name and title:**<br>**Lars Øivind Høiseth, MD, PhD**<br>Address:<br>Department of Anesthesiology<br>Division of Emergencies and Critical Care<br>Oslo University Hospital<br>P.O.Box 4956, Nydalen<br>0424 Oslo<br>[redacted]
**Investigator**:	**Name and title:**<br>**Sole Lindvåg Lie, MD**<br>Address:<br>Department of Research,<br>Norwegian Air Ambulance Foundation,<br>Postboks 414 Sentrum,<br>0103 Oslo<br>Norway<br>[redacted]

[redacted]

	[redacted]
**Investigator**:	**Name and title:**<br>**Jonny Hisdal, professor dr.philos**<br>Address:<br>Section of Vascular Investigations<br>Department of Vascular Surgery<br>Division of Cardiovascular and Pulmonary diseases<br>Oslo University Hospital<br>[redacted]
**Investigator**:	**Name and title:**<br>**Marius Rehn, MD, PhD**<br>Address:<br>Department of Research,<br>Norwegian Air Ambulance Foundation,<br>Postboks 414 Sentrum,<br>0103 Oslo<br>Norway<br>[redacted]

[redacted]

Table of Contents

1. PROTOCOL SUMMARY ... 9
1.1. Synopsis ... 9
1.2. Schema ... 11
1.3. Schedule of Activities (SoA) ... 12
2. INTRODUCTION ... 13
2.1. Study Rationale ... 13
2.2. Background ... 13
2.3. Benefit/Risk Assessment ... 14
2.3.1. Risk Assessment ... 14
2.3.2. Benefit Assessment ... 16
2.3.3. Overall Benefit: Risk Conclusion ... 16
3. OBJECTIVES AND ESTIMANDS ... 17
4. STUDY DESIGN ... 18
4.1. Overall Design ... 18
4.2. Scientific Rationale for Study Design ... 18
4.2.1. Participant Input into Design ... 18
4.3. Justification for Dose ... 19
4.4. End of Study Definition ... 19
5. STUDY POPULATION ... 20
5.1. Inclusion Criteria ... 20
5.2. Exclusion Criteria ... 21
5.3. Lifestyle Considerations ... 21
5.3.1. Meals and Dietary Restrictions ... 22
5.3.2. Caffeine, Alcohol, and Tobacco ... 22
5.3.3. Activity ... 22
5.4. Screen Failures ... 22
6. STUDY INTERVENTION(S) AND CONCOMITANT THERAPY ... 23
6.1. Study Intervention(s) Administered ... 23
6.2. Preparation/Handling/Storage/Accountability ... 23
6.3. Measures to Minimize Bias: Randomization and Blinding ... 24
6.4. Study Intervention Compliance ... 24
6.5. Dose Modification ... 25
6.6. Continued Access to Study Intervention after the End of the Study ... 25
6.7. Treatment of Overdose ... 25
6.8. Concomitant Therapy ... 25
6.8.1. Rescue Medicine ... 25
7. DISCONTINUATION OF STUDY INTERVENTION AND PARTICIPANT WITHDRAWAL ... 26
7.1. Discontinuation of Study Intervention ... 26
7.2. Participant Discontinuation/Withdrawal from the Study ... 26

7.3. Lost to Follow up 26

8. STUDY ASSESSMENTS AND PROCEDURES 27

8.1. Procedure during each visit 27
8.2. Efficacy Assessments 28
8.3. Safety Assessments 29
8.3.1. Physical Examinations 29
8.3.2. Vital Signs 29
8.3.3. Electrocardiograms 29
8.3.4. Pregnancy Testing 29
8.4. Adverse Events (AEs), Serious Adverse Events (SAEs), and Other Safety Reporting 29
8.4.1. Time Period and Frequency for Collecting AE and SAE Information 30
8.4.2. Method of Detecting AEs and SAEs 30
8.4.3. Follow-up of AEs and SAEs 30
8.4.4. Regulatory Reporting Requirements for SAEs 30
8.4.5. Pregnancy 31
8.4.6. Disease-Related Events and/or Disease-Related Outcomes Not Qualifying as AEs or SAEs 31
8.4.7. Adverse Events of Special Interest 31
8.5. Pharmacokinetics 31
8.6. Genetics 32
8.7. Biomarkers 32
8.8. Health Economics 32

9. STATISTICAL CONSIDERATIONS 33

9.1. Statistical Hypotheses 33
9.2. Sample Size Determination 33
9.3. Statistical Analyses 34
9.3.1. General Considerations 34
9.3.2. Primary Endpoint 34
9.3.3. Secondary Endpoint(s) 35
9.3.4. Tertiary/Exploratory Endpoint(s) 35
9.3.5. Safety Analysis 35
9.3.6. Other Analysis 35
9.4. Interim Analysis 35

10. SUPPORTING DOCUMENTATION AND OPERATIONAL CONSIDERATIONS 36

10.1. Appendix 1: Regulatory, Ethical, and Study Oversight Considerations 36
10.1.1. Regulatory and Ethical Considerations 36
10.1.2. Financial Disclosure 36
10.1.3. Informed Consent Process 37
10.1.4. Data Protection 37
10.1.5. Dissemination of Clinical Study Data 37
10.1.6. Data Quality Assurance 37
10.1.7. Source Documents 38
10.1.8. Study and Site Start and Closure 38
10.1.9. Publication Policy 39

[redacted]

10.2. APPENDIX 3: AEs AND SAEs: DEFINITIONS AND PROCEDURES FOR RECORDING, EVALUATING, FOLLOW-UP, AND REPORTING 40

10.2.1. Definition of AE *40*

10.2.2. Definition of SAE *41*

10.2.3. Recording and Follow-Up of AE and/or SAE *42*

10.2.4. Reporting of SAEs *44*

10.3. APPENDIX 9: ABBREVIATIONS 45

10.4. APPENDIX 10: PROTOCOL AMENDMENT HISTORY 46

11. REFERENCES **48**

[redacted]

1. Protocol Summary

1.1. Synopsis

Protocol Title:

Effects of supplemental oxygen on cerebral and systemic hemodynamics in experimental hypovolemia

A randomized, phase I, crossover study in healthy volunteers to study the effect of supplemental oxygen vs. room air on cerebral and systemic hemodynamics in healthy volunteers > 18 years during experimental hypovolemia in the lower body negative pressure model

Brief Title:

Supplemental oxygen in hypovolemia

Rationale:

Objectives and Endpoints:

Objectives	Endpoints
Primary	
• Determine if there is an effect of supplemental oxygen on cardiac output during LBNP	• The difference in the change in cardiac output (stroke volume × heart rate) between the intervention and control group during LBNP
Secondary	
• Determine if there is an effect of supplemental oxygen on cardiac stroke volume during LBNP	• The difference in the change in cardiac stroke volume between the intervention and control group during LBNP
• Determine if there is an effect of supplemental oxygen on cerebral blood flow velocity during LBNP	• The difference in the change in middle cerebral artery blood flow velocity (MCAV) between the intervention and control group during LBNP
• Determine if there is an effect of supplemental oxygen on time to hemodynamic decompensation during LBNP	• The difference in time to decompensation between the intervention and control group during LBNP

Overall Design:

[redacted]

Randomized, phase I, single-center, crossover study. Randomization performed before screening.

Intervention: Oxygen.

Comparator: Room air.

Study subjects: Healthy volunteers > 18 and < 50 years.

Blinding: Double blind.

Brief Summary:

The purpose of the study is to explore the effects of supplemental oxygen on cerebral blood flow and cardiovascular function during experimental hypovolemia in healthy volunteers. Study details include:

- Study duration: Approximately 2 hours
- Number of visits: 2
- Visit frequency: At least one day apart

Number of Participants:

Approximately 20 participants will be screened to achieve 15 subjects randomly assigned/enrolled to start with study intervention or control treatment.

Note: "Enrolled" means a participant's, or their legally acceptable representative's, agreement to participate in a clinical study following completion of the informed consent process and screening. Potential participants who are screened for the purpose of determining eligibility for the study, but do not participate in the study, are not considered enrolled, unless otherwise specified by the protocol.

Data Monitoring Committee: No

1.2. Schema

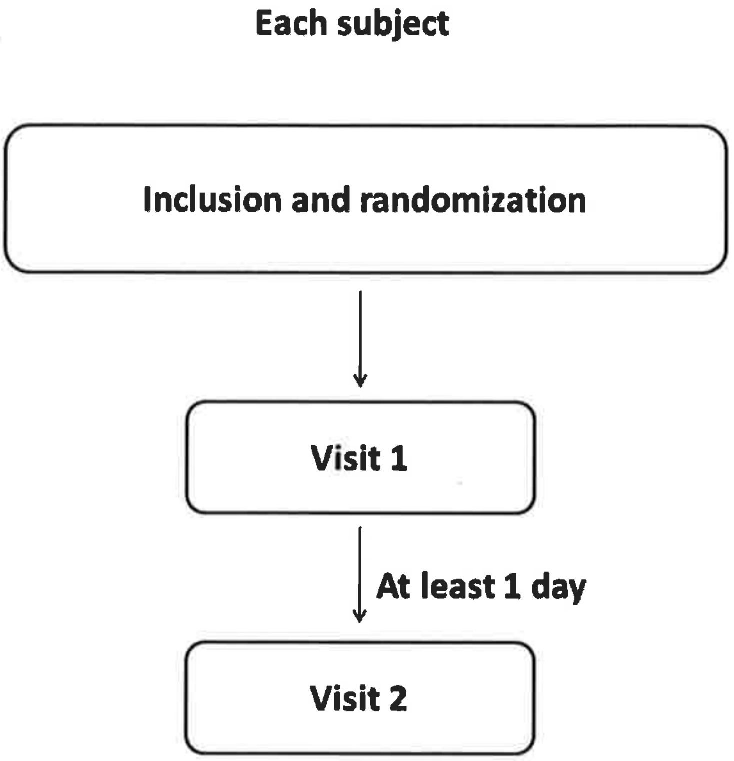

Figure 1: Timeline for each subject

1.3. Schedule of Activities (SoA)

Procedure	Screening (up to 1 day before Day 1)	Intervention Period; visit no.		E/D	Notes<br><br>E/D = Early Discontinuation
		1	2		
Informed consent	X				
Inclusion and exclusion criteria	X				Recheck clinical status before randomization and/or 1st dose of study intervention.
Demography	X				
Physical examination	X				
Medical history	X				
Past and current medical conditions	X				
Highly sensitive urine pregnancy test (WOCBP only)	X				See 8.2.5
Vital signs	X	X	X		
[Randomization] if applicable		X			
Study intervention		X	X		
AE review		←——→		X	
Unsolicited AEs		←——→		X	See Appendix 3 for definitions
SAE review		←——→		X	X
Concomitant medication review		←——→		X	X

2. Introduction

2.1. Study Rationale

Supplemental oxygen is frequently administered in acutely and critically ill patients, specifically, it is often administered in trauma patients to avoid arterial hypoxemia and tissue hypoxia. There is also an increasing focus on potentially deleterious effects of hyperoxia. Further, the hemodynamic response to hyperoxia in hypovolemia is poorly understood.

The present study aims to investigate the effects of supplemental oxygen on systemic and cerebral hemodynamics in simulated hypovolemia in healthy volunteers.

2.2. Background

The overriding goal for the resuscitation of any critically ill patient is to ensure adequate oxygen delivery. Global oxygen delivery is given by the equation [1],

$$DO_2(\frac{ml}{min}) = 10 \times CO \times (1.34 \times [Hb] \times SaO_2 \times CO + 0.0225 \times PaO_2)$$

where DO_2 is global oxygen delivery, [Hb] is hemoglobin concentration, SaO_2 is arterial oxygen saturation, CO is cardiac output and PaO_2 is arterial partial pressure of oxygen. The first term quantifies the oxygen which is hemoglobin-bound, whereas the second term quantifies the freely dissolved oxygen. Under most circumstances, the hemoglobin-bound fraction dominates quantitatively.

In critically ill patients, any factor in this equation may be reduced, thus reducing oxygen delivery. Hemoglobin concentration may be reduced due to hemorrhage, SaO_2 and PaO_2 may be reduced due to lung dysfunction (*e.g.* atelectasis and shunt flow), and CO may be reduced due to hypovolemia or cardiac dysfunction.

Under normal circumstances, SaO_2 is near to 100%, and supplemental oxygen can not increase this further. Thus, in this circumstance, providing supplemental oxygen will only increase the small, dissolved proportion. Although this proportion may be a significant fraction in some circumstances, the intention of giving supplemental oxygen is in most circumstances to ensure a high SaO_2.

Based on the above, supplemental oxygen has been extensively used in acutely critically ill patients to avoid hypoxemia, and is recommended in severely injured trauma patients, as given by the statement: "*Supplemental oxygen must be administered to all severely injured trauma patients.*" in the ATLS (Advanced Trauma Life Support) guidelines [2]. Accordingly, supplemental oxygen is often given to trauma patients, often resulting in hyperoxia [3]. The clinical evidence for providing supplemental oxygen in all trauma patients is however scarce [4].

The liberal use of supplemental oxygen has also largely been founded on a perception that supplemental oxygen is harmless, and that it is safer to err on the side of hyperoxia. There is however an increasing focus on possible deleterious effects of hyperoxia, especially in specific clinical circumstances. This has led to recommendations of more restrictive use of supplemental

oxygen, often titrated to no more than what is necessary to achieve an adequate arterial oxygen saturation (*e.g.* in the range 94-98%) [5].

In the initial treatment of trauma patients, detection and treatment of hypovolemia is of paramount importance. Hypovolemia leads to reduced cardiac filling and stroke volume [6]. Under normal circumstances in unanesthetized humans, this is compensated by an increase in systemic vascular resistance and heart rate to maintain a normal or near-normal mean arterial pressure (MAP). At some point, these compensatory mechanisms are exhausted, and MAP typically falls abruptly. The mechanisms behind this decompensation is not fully understood, but attempts have been made to estimate the degree of exhaustion of these compensator mechanisms [7].

Lower body negative pressure (LBNP) is a model of central hypovolemia where negative pressure is applied to the body from the waist-down [8]. Thereby, blood is displaced from the central compartment of the upper body to the lower extremities and pelvis. The model has been used for more than half a century and is considered useful model for studying hypovolemia in conscious volunteers.

Normobaric hyperoxia induces vasoconstriction and reduced blood flow to several organs, including the brain, heart and skeletal muscle [9,10]. One could therefore hypothesize hyperoxia leading to both an increased tolerance to hypovolemia mediated by vasoconstriction as well as a reduced tolerance mediated by reduced cerebral blood flow. One study using the LBNP-model did not find that supplemental oxygen significantly affected the hemodynamic response to simulated hypovolemia [11]. This study did however only apply one level of LBNP and did not specifically study cerebral circulation.

Based on the above, there is a need for studies on the effects of normobaric hyperoxia on the hemodynamic response to hypovolemia.

2.3. Benefit/Risk Assessment

More detailed information about the known and expected benefits and risks and reasonably expected adverse events of oxygen may be found in the Summary of Product Characteristics [12].

2.3.1. Risk Assessment

2.3.1.1. Lower body negative pressure

The lower body negative pressure (LBNP) is considered safe to use in healthy subjects [8], and we are not aware of adverse events caused by the use of the model. In our laboratory, we have extensive experience with the model. During the LBNP-exposure, the subjects are closely monitored (see 4.1). The study subjects are healthy, according to the inclusion and exclusion criteria and will undergo a focused medical examination before LBNP-exposure.

2.3.1.2. IMP; oxygen

Administration of normobaric oxygen at 100% is not recommended for >6 h due to formation of reactive oxygen species (ROS) [12] and their possible side-effects, primarily affecting the lungs

[13]. During the study, administration of 100% oxygen will in most subjects be limited to approximately 30 min, and never exceed 60 min.

In essence, in adults we are not aware of significant medical risks with the short-term use of oxygen in healthy adults. There are no absolute contraindications to normobaric oxygen supplementation [12].

Potential Risk of Clinical Significance	Summary of Data/Rationale for Risk	Mitigation Strategy
**Study Interventions**		
• Supplemental oxygen should not be given or be given with caution in the following patients [12]:<br>1. Chronic obstructive pulmonary disease.<br>2. Pediatric population.<br>3. Concurrent use of medications or chemicals possibly increasing oxygen toxicity (certain cytostatic agents, antiarrhythmic agents, antibiotics, disulfiram and paraquat).	• As presented in the SPC [12].	• Study subjects should not have any medical conditions limiting normal physical performance (as COPD), allergies exempted.<br>• Study subjects should be adults (>18 yr).<br>• Study subjects should not use any medications; medication for allergies and contraceptives exempted.
• Flammability.	• As presented in the SPC [12].	• There will be no use of open fire or electrocautery during administration of IMP.

[redacted]

**Study Procedures**		
• Lower body negative pressure, hemodynamic effects.	• As presented in reference [8]. LBNP is considered safe in healthy subjects.	• See 5.1 and 5.2. Further, the study subjects will undergo a focused ultrasound examination of the heart. During IMP-administration and LBNP exposure, at least one medical doctor and equipment for resuscitation will be present. The study visits take place in a hospital. Study subjects are < 50 years old to minimize risk of asymptomatic heart disease.
**Other**		

2.3.2. Benefit Assessment

There is no benefit for the individual study subjects by participating in the study.

Results from the study may be of benefit for critically or acutely ill hypovolemic patients, e.g. trauma patients.

2.3.3. Overall Benefit: Risk Conclusion

Taking into account the measures taken to minimize risk to participants participating in this study, the potential risks identified in association with study participation are justified by the anticipated scientific knowledge that may be gained, and the benefits this may convey for future patients, e.g. trauma patients.

[redacted]

3. Objectives and Estimands

Objectives	Estimands
Primary	
• Effect of oxygen and LNBP on changes in cardiac output.	• Regression coefficients for LBNP (continuous variable) and oxygen (factor) and their interaction effect on cardiac output.
Secondary	
• Effect of oxygen and LNBP on changes in middle cerebral artery blood flow velocity (MCAV).<br>• Effect of oxygen and LNBP on changes in cardiac stroke volume.<br>• Effect of oxygen and LNBP on changes in time to decompensation.	• Regression coefficients for LBNP (continuous variable) and oxygen (factor) and their interaction effect on MCAV.<br>• Regression coefficients for LBNP (continuous variable) and oxygen (factor) and their interaction effect on cardiac stroke volume.<br>• Time to decompensation (Kaplan-Meier plot or similar for repeated measurements) for LBNP (continuous variable) and oxygen (factor) and their interaction effect on MCAV.

4. Study Design

4.1. Overall Design

Randomized, double blind, crossover design.

Study subjects have two visits, where either oxygen of placebo (air) is given in a block-randomized order. Both study subjects and study personnel performing measurements are blinded to the treatment.

After screening, information, consent, and inclusion which takes place at the first visit, the two visits are equal. Each visit should be at least one day apart.

4.2. Scientific Rationale for Study Design

The study will be randomized and double-blinded to minimize risk of bias. The crossover design will allow each subject to serve as his/ her own control. This will increase the power of the study by reducing variability as repeated measurements within subjects are presumed to be more similar than those between subjects.

4.2.1. Scientific Rationale for Endpoints

Difference in change in cardiac output has been chosen as the primary endpoint for the study. Cardiac output is the hemodynamic variable determining global oxygen delivery which changes most during acute hemorrhage (the others being arterial oxygen saturation and hemoglobin concentration which are not expected to change to the same extent). The change in cardiac output therefore reflects the main compensatory mechanism for hemorrhage.

Cardiac stroke volume is a sensitive marker of volume loss, and together with heart rate determines cardiac output. Difference in change in stroke volume is therefore a secondary endpoint.

Middle cerebral artery blood velocity is a major determinant of cerebral blood supply. As cardiac output is the main changing determinant of global blood flow during hemorrhage, cerebral blood supply is the main determinant of cerebral oxygen delivery. As the proportion of cerebral blood flow to global blood flow may change during hemorrhage, difference in change in middle cerebral artery blood velocity has been chosen as a secondary endpoint. Time to hemodynamic decompensation represents a composite measure of the ability to compensate for hemorrhage. Difference in times to hemodynamic decompensation has therefor been chosen as a secondary endpoint.

4.2.2. Participant Input Into Design

Participants have not been consulted for this study.

[redacted]

4.3. Justification for Dose

For this device, the term "dose" refers to oxygen 15 l/min using a reservoir. This dose has been chosen as it is recommended to provide at least 10 l/min to achieve a fraction of inspired oxygen close to 1.0 in trauma patients [2].

4.4. End of Study Definition

The end of the study is defined as the date of the last visit of the last participant in the study.

A participant is considered to have completed the study if he/she has completed all phases of the study including the last visit.

[redacted]

5. Study Population

Prospective approval of protocol deviations to recruitment and enrollment criteria, also known as protocol waivers or exemptions, is not permitted.

5.1. Inclusion Criteria

Participants are eligible to be included in the study only if all of the following criteria apply:

Age

1. Participant must be 18 years of age inclusive, at the time of signing the informed consent.
2. Participant must be under 50 years of age inclusive, at the time of signing the informed consent.

Type of Participant and Disease Characteristics

3. Participants who are overtly healthy as determined by medical evaluation including medical history, heart and lung auscultation, focused cardiac ultrasound and measurement of cardiac conduction times.

Sex and Contraceptive/Barrier Requirements

4. Contraceptive use by women should be consistent with local regulations regarding the methods of contraception for those participating in clinical studies.

a. Male participants: Not applicable.
b. Female participants:

Use of adequate birth control for women of childbearing potential.

o A woman is considered of childbearing potential (WOCBP), i.e. fertile, following menarche and until becoming post-menopausal unless permanently sterile when sexually active. Permanent sterilisation methods include hysterectomy, bilateral salpingectomy and bilateral oophorectomy. A postmenopausal state is defined as no menses for 12 months without an alternative medical cause. A high follicle stimulating hormone (FSH) level in the postmenopausal range may be used to confirm a post-menopausal state in women not using hormonal contraception or hormonal replacement therapy. However, in the absence of 12 months of amenorrhea, a single FSH measurement is insufficient.

o Inclusion of WOCBP is possible when either:

- Using at least an acceptable effective contraceptive measure (combined (estrogen and progestogen containing) hormonal contraception, progestogen-only hormonal contraception associated with inhibition of

ovulation, intrauterine device, intrauterine hormone-releasing system, bilateral tubal occlusion, vasectomised partner or sexual abstinence). As a minimum contraception should be maintained until treatment discontinuation.

or

- Confirmed negative highly sensitive urine or serum pregnancy test at screening. A pregnancy test is performed at any visit before administering IMP if more than 14 days have passed since last pregnancy test. There will be no demand for post-intervention contraception.

Informed Consent

5. Capable of giving signed informed consent as described in Appendix 1 which includes compliance with the requirements and restrictions listed in the informed consent form (ICF) and in this protocol.

5.2. Exclusion Criteria

Participants are excluded from the study if any of the following criteria apply:

Medical Conditions

1. Any medical condition limiting physical exertional capacity or requiring regular medication (allergy and contraceptives excepted).
2. Pregnancy.
3. Breastfeeding.
4. History of syncope (syncope of presumed vasovagal nature with known precipitating factor excepted).
5. Any known cardiac arrhythmia.

Prior/Concomitant Therapy

6. Any drug (contraceptives excepted) used on a regular basis for a chronic condition (allergy excepted).

5.3. Lifestyle Considerations

[redacted]

5.3.1. Meals and Dietary Restrictions

Sugjects can have a light meals the day of the experiment before the experiments.

5.3.2. Caffeine, Alcohol, and Tobacco

1. During each dosing session, participants will abstain from ingesting caffeine-containing products for 6 hours before each visit.
2. Participants who use tobacco products will be instructed that use of nicotine-containing products (including nicotine patches) will not be permitted for 12 hours before each visit.

5.3.3. Activity

1. Participants will abstain from strenuous exercise for 3 hours before each visit.

5.4. Screen Failures

Screen failures are defined as participants who consent to participate in the clinical study but are not subsequently entered in the study. A minimal set of screen failure information is required to ensure transparent reporting of screen failure participants to meet the Consolidated Standards of Reporting Trials (CONSORT) publishing requirements and to respond to queries from regulatory authorities. Minimal information includes demography, screen failure details, eligibility criteria, and any serious adverse event (SAE).

Individuals who do not meet the criteria for participation in this study (screen failure) may not be rescreened.

6. Study Intervention(s) and Concomitant Therapy

Study intervention is defined as any investigational intervention(s), marketed product(s), placebo, or medical device(s) intended to be administered to a study participant according to the study protocol.

6.1. Study Intervention(s) Administered

**ARM Name**	Oxygen	Room-air
**Intervention Name**	Conoxia 100 % medisinsk gass, komprimert	Air (from central gas supply)
**Type**	Drug	Drug
**Dose Formulation**	Inhaled gas	Inhaled gas
**Unit Dose Strength(s)**	15 L/min	15 L/min
**Dosage Level(s)**	Continuous during LBNP-exposure	Continuous during LBNP-exposure
**Route of Administration**	Inhaled – mask with reservoir	Inhaled – mask with reservoir
**Use**	Experimental	Sham comparator
**IMP and NIMP**	IMP	IMP
**Sourcing**	Provided locally by the trial site.	Provided locally by the trial site.
**Packaging and Labeling**	Study Intervention will be provided in gas cylinder.	Study Intervention will be provided from central gas supply.

6.2. Preparation/Handling/Storage/Accountability

1. The investigator or designee must confirm appropriate temperature conditions have been maintained during transit for all study intervention received and any discrepancies are reported and resolved before use of the study intervention.
2. Only participants enrolled in the study may receive study intervention and only authorized site staff may supply or administer study intervention. All study intervention

must be stored in a secure, environmentally controlled, and monitored (manual or automated) area in accordance with the labeled storage conditions with access limited to the investigator and authorized site staff.

3. The investigator, institution, or the head of the medical institution (where applicable) is responsible for study intervention accountability, reconciliation, and record maintenance (i.e., receipt, reconciliation, and final disposition records).
4. Partly used or unused study interventions (oxygen cylinders) will be returned to Oslo University Hospital.

6.3. Measures to Minimize Bias: Randomization and Blinding

Study using Pre-Coded Randomization provided to site	*On Visit 1 participants will be assigned a unique number (randomization number) in ascending numerical order at each study site. The randomization number encodes the participant's assignment to one of the 2 arms of the study, according to the randomization schedule generated prior to the study.*<br>*Participants will be randomly assigned (block randomization) in a 1:1 ratio to receive study intervention or sham comparator first. To get at balanced design the participants will be randomized with permuted blocks of size 4 or 6, using the "blockrand" package in R [14]. The randomization list will be automatically generated by the principal investigator as a .pdf-document and handed to a 3rd party who will prepare hosing for oxygen or air administration (see below) and envelopes for emergency unblinding (see 8.1). Randomization lists will not be available to the investigators collecting data until after end of study. Each participant will be dispensed blinded study intervention.*

Blinded study with unblinded site personnel who is dispensing intervention	*Investigators collecting data will remain blinded to each participant's assigned study intervention throughout the course of the study. To maintain this blind, an otherwise uninvolved 3rd party will be responsible for the preparation of hosing from oxygen (IMP) or air (control) source to the study subject during the visits. Flow from both sources will be at 15 L/min during the exposure, but only one of the two will be leading to the study subject, and which source is leading to the subjects will not be visible to attending study personnel. Thereby, both the study subject and investigators collecting data will remain blinded to the assigned treatment.*

Sponsor safety staff may unblind the intervention assignment for any participant with an SAE. If the SAE requires that an expedited regulatory report be sent to one or more regulatory agencies, a copy of the report, identifying the participant's intervention assignment, may be sent to investigators in accordance with local regulations and/or sponsor policy.

6.4. Study Intervention Compliance

When participants are dosed at the site, they will receive study intervention directly from the investigator or designee, under medical supervision.

6.5. Dose Modification

No dose modification will be planned or allowed.

6.6. Continued Access to Study Intervention after the End of the Study

There will be no access to study intervention after end of the study.

6.7. Treatment of Overdose

Overdose of the IMP requires no treatment. The intended dose of IMP is the maximal achievable dose in normal atmospheric pressure, and as such, overdosing is not possible.

6.8. Concomitant Therapy

Use of any drug (contraceptives excepted) used on a regular basis for a chronic condition (allergy excepted) is an exclusion criterion.

6.8.1. Rescue Medicine

Use of a rescue medicine is not applicable for this study.

7. Discontinuation of Study Intervention and Participant Withdrawal

7.1. Discontinuation of Study Intervention

In rare instances, it may be necessary for a participant to permanently discontinue study intervention. See the SoA for data to be collected at the time of discontinuation of study intervention and follow-up and for any further evaluations that need to be completed.

If information obtained during the study indicates that the benefit/risk assessment for the study is no longer positive, the study will be discontinued.

7.2. Participant Discontinuation/Withdrawal from the Study

- A participant may withdraw from the study at any time at his/her own request or may be withdrawn at any time at the discretion of the investigator for safety, behavioral, or compliance reasons. This is expected to be uncommon.
- At the time of discontinuing from the study, if possible, an early discontinuation visit should be conducted, as shown in the SoA. See SoA for data to be collected at the time of study discontinuation and follow-up and for any further evaluations that need to be completed.
- The participant will be permanently discontinued both from the study intervention and from the study at that time.
- If the participant withdraws consent for disclosure of future information, the sponsor may retain and continue to use any data collected before such a withdrawal of consent.

7.3. Lost to Follow up

A participant will be considered lost to follow-up if he or she repeatedly fails to return for scheduled visits and is unable to be contacted by the study site.

The following actions must be taken if a participant fails to return to the clinic for a required study visit:

- The site must attempt to contact the participant and reschedule the missed visit as soon as possible and counsel the participant on the importance of maintaining the assigned visit schedule and ascertain whether or not the participant wishes to and/or should continue in the study.
- Before a participant is deemed lost to follow up, the investigator or designee must make every effort to regain contact with the participant (where possible, 3 telephone calls and, if necessary, e-mails). These contact attempts should be documented in the participant's CRF.
- Should the participant continue to be unreachable, he/she will be considered to have withdrawn from the study.

Discontinuation of specific sites or of the study as a whole are handled as part of Appendix 1.

[redacted]

8. Study Assessments and Procedures

- Study procedures and their timing are summarized in the SoA. Protocol waivers or exemptions are not allowed.
- Immediate safety concerns should be discussed with the sponsor immediately upon occurrence or awareness to determine if the participant should continue or discontinue study intervention.
- Adherence to the study design requirements, including those specified in the SoA, is essential and required for study conduct.
- All screening evaluations must be completed and reviewed to confirm that potential participants meet all eligibility criteria. The investigator will maintain a screening log to record details of all participants screened and to confirm eligibility or record reasons for screening failure, as applicable.
- Procedures conducted as part of the participant's routine clinical management (e.g., blood count) and obtained before signing of the ICF may be utilized for screening or baseline purposes provided the procedures met the protocol-specified criteria and were performed within the time frame defined in the SoA.
- Analyte results that could unblind the study will not be reported to investigative sites or other blinded personnel until the study has been unblinded.
- Any changes as given by the medical history and/ or vital signs relevant to continued participation in the study are considered.

8.1. Procedure during each visit

At each visit, monitoring equipment is attached (see 8.2). Mean arterial pressure and heart rate are measured at baseline to determine *stop-criteria* (see below).

Administration of IMP starts. Run-in 5 min.

LBNP-exposure starts with 3 min LBNP 0 mmHg, followed by LNBP 10 mmHg for 3 min and further stepwise increases in LBNP of 10 mmHg, each step lasting 3 min.

In the unlikely case of discontinuation of study intervention due to medical considerations, investigators will carry out emergency unblinding by opening an envelope present at all visits containing the given treatment. This envelope will be shredded after end of visit.

LBNP is released after 3 min at LBNP 80 mmHg or sooner by occurrence of any of the following criteria:

- Symptoms or signs of impending circulatory collapse
  - Symptoms of pre-syncope (light-headedness, nausea or sweating)
  - Occurrence of hemodynamic stop-criteria preceding circulatory collapse (determined from measurements at baseline – see above)
    - MAP-reduction to less than 75% of baseline values (measured at normovolemia) for >3 s

- HR-reduction to less than 75% baseline values (measured at normovolemia) for >3 s

- Subject request for reasons other than above

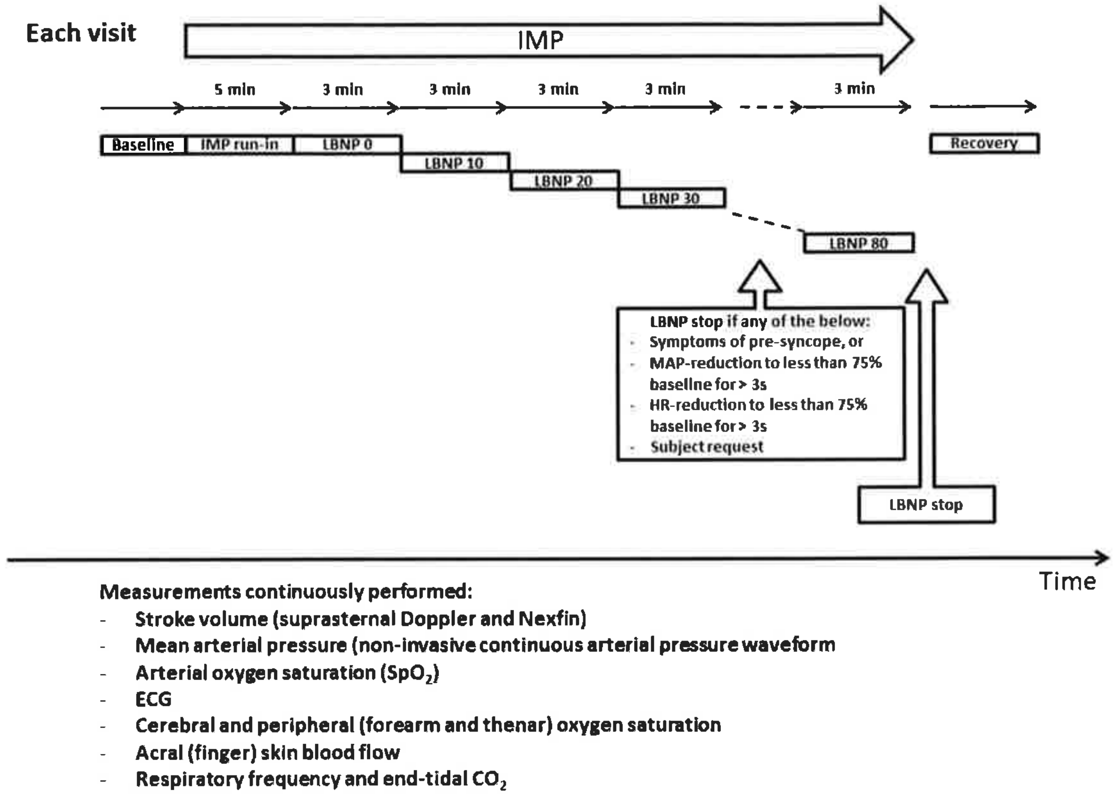

Figure 2: Timeline for each visit.

8.2. Efficacy Assessments

The following variables will be recorded during LBNP.

- - Mean arterial pressure and stroke volume: Volume clamp (Nexfin; Edwards Lifesciences corp., CA, USA)
- - Stroke volume. Suprasternal Doppler (SD-50 (SD-50; Vingmed Ultrasound, Horten, Norway)
- MCAV (GE E95; General Electric/ Vingmed, Horten, Norway)
- - Arterial oxygen saturation: Pulse oximetry (Masimo Radical 7; Maximo corp., CA, USA)

- - ECG (Powerlab; ADInstruments, Dunedin, New Zealand)
- - Cerebral and somatic (forearm and thenar) oxygen saturation: Near infra-red spectroscopy (Invos 5100C cerebral/somatic oximeter; Somanetics, Troy, MI, USA)
- - Acral (finger) skin blood flow: Laser Doppler flowmetry (PeriFlux 4001 Master; Perimed AB, Järfälla, Sweden)
- - Respiratory frequency and end-tidal CO_2 : Volumetric capnography (Medlab CAP 10; Medlab GmbH, Stutensee, Germany)

8.3. Safety Assessments

Planned time points for all safety assessments are provided in the SoA.

8.3.1. Physical Examinations

- A complete physical examination will include, at a minimum, assessments of the cardiovascular and respiratory systems. Height and weight will also be measured and recorded.
- A brief physical examination will include, at a minimum, assessments of the lungs, and cardiovascular system.
- Investigators should pay special attention to clinical signs related to previous serious illnesses.

8.3.2. Vital Signs

- Pulse rate, respiratory rate, and blood pressure will be assessed.
- Blood pressure and pulse measurements will be assessed supine with a completely automated device.
- Blood pressure and pulse measurements should be preceded by at least 5 minutes of rest for the participant in a quiet setting without distractions (e.g., television, cell phones).

8.3.3. Electrocardiograms

- Three-lead ECG will be obtained to screen for conduction abnormalities as outlined in the SoA (see Section 1.3) using an ECG machine that automatically calculates the heart rate and measures PR. PQ, QRS and QT-times are manually measured.

8.3.4. Pregnancy Testing

See 5.1 and 5.2.

8.4. Adverse Events (AEs), Serious Adverse Events (SAEs), and Other Safety Reporting

The definitions of adverse events (AEs) and serious adverse events (SAEs) can be found in Appendix 3.

The definitions of unsolicited and solicited adverse events can be found in Appendix 3.

AEs will be reported by the participant.

The investigator and any qualified designees are responsible for detecting, documenting, and recording events that meet the definition of an AE or SAE and remain responsible for following up all AEs OR AEs that are serious, considered related to the study intervention or study procedures, or that caused the participant to discontinue the study (see Section 7).

The method of recording, evaluating, and assessing causality of AEs and SAEs and the procedures for completing and transmitting SAE reports are provided in Appendix 3.

8.4.1. Time Period and Frequency for Collecting AE and SAE Information

All AEs and SAEs will be collected from the start of intervention until end of the last visit at the time points specified in the SoA (Section 1.3).

Medical occurrences that begin before the start of study intervention but after obtaining informed consent will be recorded as Medical History/Current Medical Conditions, not as AEs.

All SAEs will be recorded and reported to the sponsor or designee immediately and under no circumstance should this exceed 24 hours, as indicated in Appendix 3. The investigator will submit any updated SAE data to the sponsor within 24 hours of it being available.

Investigators are not obligated to actively seek information on AEs or SAEs after conclusion of the study participation. However, if the investigator learns of any SAE, including a death, at any time after a participant has been discharged from the study, and he/she considers the event to be reasonably related to the study intervention or study participation, the investigator must promptly notify the sponsor.

Fatal or life threatening SUSARs will be reported to The Norwegian Medicines Agency within 7 days, and other SUSARs within 15 days.

8.4.2. Method of Detecting AEs and SAEs

Care will be taken not to introduce bias when detecting AEs and/or SAEs. Open-ended and non-leading verbal questioning of the participant is the preferred method to inquire about AE occurrences.

8.4.3. Follow-up of AEs and SAEs

After the initial AE/SAE report, the investigator is required to proactively follow each participant at subsequent visits/contacts. All SAEs, will be followed until resolution, stabilization, the event is otherwise explained, or the participant is lost to follow-up (as defined in Section 7.3). Further information on follow-up procedures is provided in Appendix 3.

8.4.4. Regulatory Reporting Requirements for SAEs

- Prompt notification by the investigator to the sponsor of an SAE is essential so that legal obligations and ethical responsibilities towards the safety of participants and the safety of a study intervention under clinical investigation are met.

- The sponsor has a legal responsibility to notify both the local regulatory authority and other regulatory agencies about the safety of a study intervention under clinical investigation. The sponsor will comply with country-specific regulatory requirements relating to safety reporting to the regulatory authority, Institutional Review Boards (IRB)/Independent Ethics Committees (IEC), and investigators.
- Investigator safety reports must be prepared for suspected unexpected serious adverse reactions (SUSAR) according to local regulatory requirements and sponsor policy and forwarded to investigators as necessary.

8.4.5. Pregnancy

See 5.1 and 5.2.

- Details of all pregnancies in female participants will be collected after the start of study intervention and until end of last visit.
- If a pregnancy is reported, the investigator will record pregnancy information on the appropriate form and submit it to the sponsor within 24 hours of learning of the female participant pregnancy.
- While pregnancy itself is not considered to be an AE or SAE, any pregnancy complication or elective termination of a pregnancy for medical reasons will be reported as an AE or SAE.
- Abnormal pregnancy outcomes (e.g., spontaneous abortion, fetal death, stillbirth, congenital anomalies, ectopic pregnancy) are considered SAEs and will be reported as such.
- The participant will be followed to determine the outcome of the pregnancy. The investigator will collect follow-up information on the participant and the neonate and the information will be forwarded to the sponsor.
- Any post-study pregnancy-related SAE considered reasonably related to the study intervention by the investigator will be reported to the sponsor as described in Section 8.4.4. While the investigator is not obligated to actively seek this information in former study participants, he or she may learn of an SAE through spontaneous reporting.
- Any female participant who becomes pregnant while participating in the study will be withdrawn from the study.

8.4.6. Disease-Related Events and/or Disease-Related Outcomes Not Qualifying as AEs or SAEs

Not applicable.

8.4.7. Adverse Events of Special Interest

Not applicable.

8.5. Pharmacokinetics

- PK parameters are not evaluated in this study.

[redacted]

8.6. Genetics

Genetics are not evaluated in this study.

8.7. Biomarkers

Biomarkers are not evaluated in this study.

8.8. Health Economics

Health Economics are not evaluated in this study.

[redacted]

9. Statistical Considerations

9.1. Statistical Hypotheses

6. Primary objective:
   - o Effect of oxygen and LNBP on changes in cardiac output.
     - H_0: There is no effect of oxygen on the change in cardiac output with LBNP.
     - H_A: There is an effect of oxygen on the change in cardiac output with LBNP.
7. Secondary objectives:
   - o • Effect of oxygen and LNBP on changes in cardiac stroke volume.
     - H_0: There is no effect of oxygen on the change in stroke volume with LBNP.
     - H_A: There is an effect of oxygen on the change in stroke volume with LBNP.
   - o • Effect of oxygen and LNBP of changes in middle cerebral artery blood flow velocity (MCAV).
     - H_0: There is no effect of oxygen on the change in MCAV with LBNP.
     - H_A: There is an effect of oxygen on the change in MCAV with LBNP.
   - o • Effect of oxygen and LNBP on time to decompensation.
     - H_0: There is no effect of oxygen on the time to decompensation with LBNP.
     - H_A: There is an effect of oxygen on the time to decompensation with LBNP.

9.2. Sample Size Determination

Approximately 20 participants will be screened to achieve 15 subjects enrolled.

Note: "Enrolled" means a participant's, or their legally acceptable representative's, agreement to participate in a clinical study following completion of the informed consent process and screening. Potential participants who are screened for the purpose of determining eligibility for the study, but do not participate in the study, are not considered enrolled, unless otherwise specified by the protocol.

The estimated effect of LBNP on cardiac output with its standard deviation was estimated from a previous experiment [15]. A reduction of 15% in cardiac output is often used as a threshold when evaluating interventions to increase cardiac output [16]. Assuming a mean cardiac output of 4.85 ± 1.08 l/min at baseline, and a change of -0.489 for each LBNP-level (each level -20 mmHg). Error within subjects is assumed independent between LBNP-levels with SD 0.385 l/min. Assuming that a 15% reduction of oxygen compared to air is significant, this would give an increased reduction (interaction effect) of 0.18 l/min per LBNP-level. If assuming a SD of 0.18 l/min for this interaction effect, including 15 subjects would give a $1-\beta=0.87$ to detect this effect with $\alpha=0.05$.

[redacted]

For the purposes of analysis, the following analysis sets are defined:

Participant Analysis Set	***Description***
Randomized	
Evaluable	
Safety	*[All randomized participants who are exposed to study intervention. Participants will be analyzed according to the intervention they actually received].*

Defined Analysis Data Sets	***Description***
Analysis set for primary estimand	*All randomized participants who are exposed to study intervention.*
Analysis set for secondary estimands	*All randomized participants who are exposed to study intervention.*

9.3. Statistical Analyses

The statistical analysis plan will be finalized prior to un-blinding and it will include a more technical and detailed description of the statistical analyses described in this section. This section is a summary of the planned statistical analyses of the most important endpoints including primary and key secondary endpoints.

9.3.1. General Considerations

Analogue data will be sampled as waveforms at 1000 Hz. They will further be downsampled beat-by-beat (heartbeats), calculating stroke volume and heart rate for each cardiac cycle. Trimmed means (trimming e.g. 5 % highest and lowest values to remove erroneous values) for each LBNP-level will be calculated. Effects of treatment group (oxygen vs. placebo) will be assessed in linear mixed-effects regression models with subjects as random effects and treatment and LBNP-level or time as fixed effects. P-values < 0.05 will be considered statistically significant. All significance tests will be two-sided.

9.3.2. Primary Endpoint

Cardiac output will be measured continuously by suprasternal Doppler and volume-clamp method if needed (e.g. due to poor signal quality). The primary aim of the study is to test the effect of oxygen compared to placebo on changes in cardiac output. P-values <0.05 are considered statistically significant. Cardiac output will be entered as a continuous explanatory variable in a linear mixed effects regression model. Analyses will be performed by assigning variables (dummy coding) to each medication. Confidence intervals for the effect of each medication will be calculated using the *glht*-function of the “multcomp”-package in R with

[redacted]

“single step” correction for multiple comparisons. No interim-analyses will be performed. Missing values will not be imputed, but are generally handled well in mixed effects regression models. The exact setup of the statistical model (e.g. random intercept vs. random slope, interaction effects, covariance structures etc.) will depend on details in the data. Analyses will be performed using the statistical software R (https://www.r-project.org/).

9.3.3. Secondary Endpoint(s)

- Change in MCAV with increasing LBNP will be analyzed in a linear mixed effects model corresponding to cardiac output (see above).

- Change in stroke volume with increasing LBNP will be analyzed in a linear mixed effects model corresponding to cardiac output (see above).

- Time to hemodynamic decompensation will be compared between the three interventions in a mixed proportional hazards model (Cox regression).

9.3.4. Tertiary/Exploratory Endpoint(s)

9.3.5. Safety Analysis

The safety analyses population will include all subjects who have received any study treatment.

9.3.6. Other Analysis

In a convenience-sampled subgroup, measurements of internal carotid artery blood velocity (ICAV) will be measured to compare with cardiac output and MCAV.

Exploratory analyses will be performed for other measurements performed during the study both related to the primary endpoint of the present study as well as other, related research questions relating to hypovolemia and hemodynamics.

9.4. Interim Analysis

No interim analysis is planned.

10. Supporting Documentation and Operational Considerations

10.1. Appendix 1: Regulatory, Ethical, and Study Oversight Considerations

10.1.1. Regulatory and Ethical Considerations

- This study will be conducted in accordance with the protocol and with the following:
  - Consensus ethical principles derived from international guidelines including the Declaration of Helsinki and Council for International Organizations of Medical Sciences (CIOMS) International Ethical Guidelines
  - Applicable ICH Good Clinical Practice (GCP) Guidelines
  - Applicable laws and regulations
- The protocol, protocol amendments, ICF, Investigator Brochure, and other relevant documents (e.g., advertisements) must be submitted to an IRB/IEC by the investigator and reviewed and approved by the IRB/IEC before the study is initiated.
- Any amendments to the protocol will require IRB/IEC approval before implementation of changes made to the study design, except for changes necessary to eliminate an immediate hazard to study participants.
- Protocols and any substantial amendments to the protocol will require health authority approval prior to initiation except for changes necessary to eliminate an immediate hazard to study participants.
- The investigator will be responsible for the following:
  - Providing written summaries of the status of the study to the IRB/IEC annually or more frequently in accordance with the requirements, policies, and procedures established by the IRB/IEC
  - Notifying the IRB/IEC of SAEs or other significant safety findings as required by IRB/IEC procedures
  - Providing oversight of the conduct of the study at the site and adherence to requirements of 21 CFR, ICH guidelines, the IRB/IEC, European regulation 536/2014 for clinical studies (if applicable), European Medical Device Regulation 2017/745 for clinical device research (if applicable), and all other applicable local regulations

10.1.2. Financial Disclosure

Investigators and sub-investigators will provide the sponsor with sufficient, accurate financial information as requested to allow the sponsor to submit complete and accurate financial certification or disclosure statements to the appropriate regulatory authorities. Investigators are responsible for providing information on financial interests during the course of the study and for 1 year after completion of the study.

The study will be funded by the Norwegian Air Ambulance Foundation and Oslo University Hospital.

10.1.3. Informed Consent Process

- The investigator or his/her representative will explain the nature of the study to the participant and answer all questions regarding the study.
- Participants must be informed that their participation is voluntary. Participants will be required to sign a statement of informed consent that meets the requirements of 21 CFR 50, local regulations, ICH guidelines, Health Insurance Portability and Accountability Act (HIPAA) requirements, where applicable, and the IRB/IEC or study center.
- Participants must be re-consented to the most current version of the ICF(s) during their participation in the study.
- A copy of the ICF(s) must be provided to the participant.

10.1.4. Data Protection

- Participants will be assigned a unique identifier by the sponsor. Any participant records or datasets that are transferred to the sponsor will contain the identifier only; participant names or any information which would make the participant identifiable will not be transferred.
- The participant must be informed that his/her personal study-related data will be used by the sponsor in accordance with local data protection law. The level of disclosure must also be explained to the participant who will be required to give consent for their data to be used as described in the informed consent

10.1.5. Dissemination of Clinical Study Data

10.1.6. Data Quality Assurance

- All participant data relating to the study will be recorded on printed or electronic CRF unless transmitted to the sponsor or designee electronically (e.g., laboratory data). The investigator is responsible for verifying that data entries are accurate and correct by physically or electronically signing the CRF.
- The investigator must permit study-related monitoring, audits, IRB/IEC review, and regulatory agency inspections and provide direct access to source data documents.
- Monitoring details describing strategy (e.g., risk-based initiatives in operations and quality such as Risk Management and Mitigation Strategies and Analytical Risk-Based Monitoring), methods, responsibilities and requirements, including handling of noncompliance issues and monitoring techniques (central, remote, or on-site monitoring) are provided in the Monitoring Plan.
- The sponsor or designee is responsible for the data management of this study including quality checking of the data.
- The sponsor assumes accountability for actions delegated to other individuals (e.g., Contract Research Organizations).
- Records and documents, including signed ICFs, pertaining to the conduct of this study must be retained by the investigator for 15 years after study completion unless local regulations or institutional policies require a longer retention period. No records may be

[redacted]

destroyed during the retention period without the written approval of the sponsor. No records may be transferred to another location or party without written notification to the sponsor.

10.1.7. Source Documents

- Source documents provide evidence for the existence of the participant and substantiate the integrity of the data collected. Source documents are filed at the investigator's site.
- Data reported on the CRF or entered in the eCRF that are transcribed from source documents must be consistent with the source documents or the discrepancies must be explained. The investigator may need to request previous medical records or transfer records, depending on the study. Also, current medical records must be available.
- The investigator must maintain accurate documentation (source data) that supports the information entered in the CRF.
- Study monitors will perform ongoing source data verification to confirm that data entered into the CRF by authorized site personnel are accurate, complete, and verifiable from source documents; that the safety and rights of participants are being protected; and that the study is being conducted in accordance with the currently approved protocol and any other study agreements, ICH GCP, and all applicable regulatory requirements.

10.1.8. Study and Site Start and Closure

First Act of Recruitment

The study start date is the date on which the clinical study will be open for recruitment of participants.

Study/Site Termination

The sponsor or designee reserves the right to close the study site or terminate the study at any time for any reason at the sole discretion of the sponsor. Study sites will be closed upon study completion. A study site is considered closed when all required documents and study supplies have been collected and a study-site closure visit has been performed.

The investigator may initiate study-site closure at any time, provided there is reasonable cause and sufficient notice is given in advance of the intended termination.

Reasons for the early closure of a study site by the sponsor or investigator may include but are not limited to:

For study termination:

- Discontinuation of further study intervention development
- Failure of the investigator to comply with the protocol, the requirements of the IRB/IEC or local health authorities, the sponsor's procedures, or GCP guidelines
- Inadequate or no recruitment (evaluated after a reasonable amount of time) of participants by the investigator
- Total number of participants included earlier than expected

[redacted]

If the study is prematurely terminated or suspended, the sponsor shall promptly inform the investigators, the IECs/IRBs, the regulatory authorities, and any contract research organization(s) used in the study of the reason for termination or suspension, as specified by the applicable regulatory requirements. The investigator shall promptly inform the participant and should assure appropriate participant therapy and/or follow-up

10.1.9. Publication Policy

- The results of this study may be published or presented at scientific meetings. If this is foreseen, the investigator agrees to submit all manuscripts or abstracts to the sponsor before submission. This allows the sponsor to protect proprietary information and to provide comments.
- The sponsor will comply with the requirements for publication of study results. In accordance with standard editorial and ethical practice, the sponsor will generally support publication of multicenter studies only in their entirety and not as individual site data. In this case, a coordinating investigator will be designated by mutual agreement.
- Authorship will be determined by mutual agreement and in line with International Committee of Medical Journal Editors authorship requirements.

10.2. Appendix 3: AEs and SAEs: Definitions and Procedures for Recording, Evaluating, Follow-up, and Reporting

10.2.1. Definition of AE

**AE Definition**
• An AE is any untoward medical occurrence in a clinical study participant, temporally associated with the use of study intervention, whether or not considered related to the study intervention.<br>• NOTE: An AE can therefore be any unfavorable and unintended sign (including an abnormal laboratory finding), symptom, or disease (new or exacerbated) temporally associated with the use of study intervention.
**Definition of Unsolicited and Solicited AE**
• An unsolicited adverse event is an adverse event that was not solicited using a Participant Diary and that is communicated by a participant who has signed the informed consent. Unsolicited AEs include serious and non-serious AEs.<br>• Potential unsolicited AEs may be medically attended (i.e., symptoms or illnesses requiring a hospitalisation, or emergency room visit, or visit to/by a health care provider). The participants will be instructed to contact the site as soon as possible to report medically attended event(s), as well as any events that, though not medically attended, are of participant concern. Detailed information about reported unsolicited AEs will be collected by qualified site personnel and documented in the participant's records.<br>• Unsolicited AEs that are not medically attended nor perceived as a concern by participant will be collected during interview with the participants and by review of available medical records at the next visit.<br>• Solicited AEs are predefined local and systemic events for which the participant is specifically questioned, and which are noted by the participant in their diary.
**Events Meeting the AE Definition**
• Any abnormal laboratory test results (hematology, clinical chemistry, or urinalysis) or other safety assessments (e.g., ECG, radiological scans, vital signs measurements), including those that worsen from baseline, considered clinically significant in the medical and scientific judgment of the investigator (i.e., not related to progression of underlying disease).<br>• Exacerbation of a chronic or intermittent pre-existing condition including either an increase in frequency and/or intensity of the condition.<br>• New conditions detected or diagnosed after study intervention administration even though it may have been present before the start of the study.<br>• Signs, symptoms, or the clinical sequelae of a suspected intervention- intervention interaction.

- Signs, symptoms, or the clinical sequelae of a suspected overdose of either study intervention or a concomitant medication. Overdose per se will not be reported as an AE/SAE unless it is an intentional overdose taken with possible suicidal/self-harming intent. Such overdoses should be reported regardless of sequelae.
- The signs, symptoms, and/or clinical sequelae resulting from lack of efficacy will be reported as AE or SAE if they fulfill the definition of an AE or SAE. "Lack of efficacy" or "failure of expected pharmacological action" also constitutes an AE or SAE.

Events NOT Meeting the AE Definition

- Any clinically significant abnormal laboratory findings or other abnormal safety assessments which are associated with the underlying disease, unless judged by the investigator to be more severe than expected for the participant's condition.
- The disease/disorder being studied or expected progression, signs, or symptoms of the disease/disorder being studied, unless more severe than expected for the participant's condition.
- Medical or surgical procedure (e.g., endoscopy, appendectomy): the condition that leads to the procedure is the AE.
- Situations in which an untoward medical occurrence did not occur (social and/or convenience admission to a hospital).
- Anticipated day-to-day fluctuations of pre-existing disease(s) or condition(s) present or detected at the start of the study that do not worsen.
- Symptoms normally experienced during LBNP at decompensation/ near-syncope (light-headedness, grey-out, transient bradycardia and hypotension).

10.2.2. Definition of SAE

An SAE is defined as any serious adverse event that, at any dose:

a. Results in death

b. Is life-threatening

The term 'life-threatening' in the definition of 'serious' refers to an event in which the participant was at risk of death at the time of the event. It does not refer to an event, which hypothetically might have caused death, if it were more severe.

c. Requires inpatient hospitalization or prolongation of existing hospitalization

- In general, hospitalization signifies that the participant has been admitted (usually involving at least an overnight stay) at the hospital or emergency ward for observation and/or treatment that would not have been appropriate in the physician's office or outpatient setting. Complications that occur during hospitalization are AEs. If a complication prolongs hospitalization or fulfills any other serious criteria, the event is

serious. When in doubt as to whether "hospitalization" occurred or was necessary, the AE should be considered serious.

- Hospitalization for elective treatment of a pre-existing condition that did not worsen from baseline is not considered an AE.

d. Results in persistent or significant disability/incapacity

- The term disability means a substantial disruption of a person's ability to conduct normal life functions.
- This definition is not intended to include experiences of relatively minor medical significance such as uncomplicated headache, nausea, vomiting, diarrhea, influenza, and accidental trauma (e.g., sprained ankle) which may interfere with or prevent everyday life functions but do not constitute a substantial disruption.

e. Is a congenital anomaly/birth defect

f. Other situations:

- Medical or scientific judgment should be exercised by the investigator in deciding whether SAE reporting is appropriate in other situations such as significant medical events that may jeopardize the participant or may require medical or surgical intervention to prevent one of the other outcomes listed in the above definition. These events should usually be considered serious.
    - Examples of such events include invasive or malignant cancers, intensive treatment for allergic bronchospasm, blood dyscrasias, convulsions or development of intervention dependency or intervention abuse.

10.2.3. Recording and Follow-Up of AE and/or SAE

AE and SAE Recording

- When an AE/SAE occurs, it is the responsibility of the investigator to review all documentation (e.g., hospital progress notes, laboratory reports, and diagnostics reports) related to the event.
- The investigator will then record all relevant AE/SAE information.
- It is **not** acceptable for the investigator to send photocopies of the participant's medical records to sponsor in lieu of completion of the CIOMS form.
- The investigator will attempt to establish a diagnosis of the event based on signs, symptoms, and/or other clinical information. Whenever possible, the diagnosis (not the individual signs/symptoms) will be documented as the AE/SAE.

Assessment of Intensity

The investigator will make an assessment of intensity for each AE and SAE reported during the study and assign it to 1 of the following categories:

[redacted]

- Mild: An event that is easily tolerated by the participant, causing minimal discomfort and not interfering with everyday activities.
- Moderate: An event that causes sufficient discomfort to interfere with normal everyday activities.
- Severe: An event that prevents normal everyday activities. An AE that is assessed as severe should not be confused with an SAE. Severe is a category utilized for rating the intensity of an event; and both AEs and SAEs can be assessed as severe.

  An event is defined as 'serious' when it meets at least 1 of the predefined outcomes as described in the definition of an SAE, NOT when it is rated as severe.

Assessment of Causality

- The investigator is obligated to assess the relationship between study intervention and each occurrence of each AE/SAE.
- A "reasonable possibility" of a relationship conveys that there are facts, evidence, and/or arguments to suggest a causal relationship, rather than a relationship cannot be ruled out.
- The investigator will use clinical judgment to determine the relationship.
- Alternative causes, such as underlying disease(s), concomitant therapy, and other risk factors, as well as the temporal relationship of the event to study intervention administration will be considered and investigated.
- The investigator will also consult the Investigator's Brochure (IB) and/or Product Information, for marketed products, in his/her assessment.
- For each AE/SAE, the investigator **must** document in the medical notes that he/she has reviewed the AE/SAE and has provided an assessment of causality.
- There may be situations in which an SAE has occurred and the investigator has minimal information to include in the initial report to sponsor. However, it is very important that the investigator always make an assessment of causality for every event before the initial transmission of the SAE data to sponsor.
- The investigator may change his/her opinion of causality in light of follow-up information and send an SAE follow-up report with the updated causality assessment.
- The causality assessment is one of the criteria used when determining regulatory reporting requirements.

Follow-up of AEs and SAEs

- The investigator is obligated to perform or arrange for the conduct of supplemental measurements and/or evaluations as medically indicated or as requested by sponsor to elucidate the nature and/or causality of the AE or SAE as fully as possible. This may

include additional laboratory tests or investigations, histopathological examinations, or consultation with other health care professionals.<br>• If a participant dies during participation in the study or during a recognized follow-up period, the investigator will provide sponsor with a copy of any post-mortem findings including histopathology.<br>• New or updated information will be recorded in the originally submitted documents.<br>• The investigator will submit any updated SAE data to sponsor within 24 hours of receipt of the information.

10.2.4. Reporting of SAEs

**SAE Reporting to sponsor via an Electronic Data Collection Tool**
• The primary mechanism for reporting an SAE to sponsor will be the electronic data collection tool.<br>• If the electronic system is unavailable, then the site will use the paper SAE data collection tool (see next section) to report the event within 24 hours.<br>• The site will enter the SAE data into the electronic system as soon as it becomes available.<br>• After the study is completed at a given site, the electronic data collection tool will be taken off-line to prevent the entry of new data or changes to existing data.<br>• SUSARs will be reported to the Competent Authority (The Norwegian Medicines Agency and REK sør-øst) according to national regulation.

**SAE Reporting to sponsor via Paper Data Collection Tool**
• Facsimile transmission of the SAE paper data collection tool is the preferred method to transmit this information to the sponsor.

[redacted]

10.3. Appendix 9: Abbreviations

MCAV	middle cerebral artery velocity
CCAV	common carotid artery velocity
ICAV	internal carotid artery velocity

[redacted]

10.4. Appendix 10: Protocol Amendment History

The Protocol Amendment Summary of Changes Table for the current amendment is located directly before the Table of Contents (TOC).

Amendment 1: 06-08-2021 Protocol Number: 2_06-08-2021

This amendment is considered to be substantial based on the criteria set forth in Article 10(a) of Directive 2001/20/EC of the European Parliament and the Council of the European Union.

Overall Rationale for the Amendment

Section # and Name	Description of Change	Brief Rationale
5.2; Exclusion criteria	Criteria 7-9 removed	Nor-applicable criteria removed after request *Statens legemiddelverk*
1.1 and 9.3.2; Endpoints	Precision made in endpoints as *differences in changes* in the mentioned variables.	After request *Statens legemiddelverk*
4.2.1; Scientific rationale for endpoints	Scientific rationale for endpoints added.	After request *Statens legemiddelverk*
5.1; Inclusion criteria	Change in criteria for inclusion of WOCBP.	After request *Statens legemiddelverk*
6.3; Measures to minimize bias: Randomization and blinding	Precision made in descriptions for randomization and blinding	After request *Statens legemiddelverk*
8.4.1; Time Period and Frequency for Collecting AE and SAE Information	Deadlines for reporting of SUSARS have been added.	After request *Statens legemiddelverk*
8.1; Procedure during each visit	Procedure for emergency unblinding have been added.	After request *Statens legemiddelverk*
7.1; Discontinuation of study intervention	Criteria for discontinuation of study based on risk-benefit assessment have been added.	After request *Statens legemiddelverk*

[redacted]

Amendment 2: 01-10-2021 Protocol Number: 3_01-10-2021

Section # and Name	Description of Change	Brief Rationale
5.1 Inclusion criteria	Upper age limit changed to 50 years	After request Regional Ethics Committee. To reduce risk of asymptomatic heart disease.
8.1. Procedure during each visit	Precisions made in criteria to abort LBNP-exposure	After request Regional Ethics Committee.

Amendment 3: 14-12-2021 Protocol Number: 4_14-12-2021

Section # and Name	Description of Change	Brief Rationale
Medical monitor	Changed medical monitor	After request by Clinical Trials Unit.

[redacted]

11. References

1. Dunn, J.-O.; Mythen, M.; Grocott, M. Physiology of Oxygen Transport. *BJA Education* **2016**, *16*, 341–348, doi:10.1093/bjaed/mkw012.

2. Trauma, C. on *ATLS Advanced Trauma Life Support 10th Edition Student Course Manual*; ACS American College of Surgeons: Place of publication not identified, 2018; ISBN 978-0-9968262-3-5.

3. Eskesen, T.G.; Baekgaard, J.S.; Christensen, R.E.; Lee, J.M.; Velmahos, G.C.; Steinmetz, J.; Rasmussen, L.S. Supplemental Oxygen and Hyperoxemia in Trauma Patients: A Prospective, Observational Study. *Acta Anaesthesiol Scand* **2019**, *63*, 531–536, doi:10.1111/aas.13301.

4. Eskesen, T.G.; Baekgaard, J.S.; Steinmetz, J.; Rasmussen, L.S. Initial Use of Supplementary Oxygen for Trauma Patients: A Systematic Review. *BMJ Open* **2018**, *8*, e020880, doi:10.1136/bmjopen-2017-020880.

5. O'Driscoll, B.R.; Howard, L.S.; Earis, J.; Mak, V. BTS Guideline for Oxygen Use in Adults in Healthcare and Emergency Settings. *Thorax* **2017**, *72*, ii1–ii90, doi:10.1136/thoraxjnl-2016-209729.

6. Vincent, J.-L.; De Backer, D. Circulatory Shock. *New England Journal of Medicine* **2013**, *369*, 1726–1734.

7. Suresh, M.R.; Chung, K.K.; Schiller, A.M.; Holley, A.B.; Howard, J.T.; Convertino, V.A. Unmasking the Hypovolemic Shock Continuum: The Compensatory Reserve. *J Intensive Care Med* **2019**, *34*, 696–706, doi:10.1177/0885066618790537.

8. Goswami, N.; Blaber, A.P.; Hinghofer-Szalkay, H.; Convertino, V.A. Lower Body Negative Pressure: Physiological Effects, Applications, and Implementation. *Physiological reviews* **2019**, *99*, 807–851.

9. Brugniaux, J.V.; Coombs, G.B.; Barak, O.F.; Dujic, Z.; Sekhon, M.S.; Ainslie, P.N. Highs and Lows of Hyperoxia: Physiological, Performance, and Clinical Aspects. *Am J Physiol Regul Integr Comp Physiol* **2018**, *315*, R1–R27, doi:10.1152/ajpregu.00165.2017.

10. Smit, B.; Smulders, Y.M.; van der Wouden, J.C.; Oudemans-van Straaten, H.M.; Spoelstra-de Man, A.M.E. Hemodynamic Effects of Acute Hyperoxia: Systematic Review and Meta-Analysis. *Critical Care* **2018**, *22*, 45, doi:10.1186/s13054-018-1968-2.

11. Kim, Y.K.; Jun, I.G.; Kim, S.R.; Hwang, J.H.; Cho, S.K.; Han, S.M.; Hwang, G.S. Using 100% Oxygen Does Not Alter the Cardiovascular Autonomic Regulation during Non-Invasively Simulated Haemorrhage in Healthy Volunteers. *J Int Med Res* **2008**, *36*, 227–236, doi:10.1177/147323000803600203.

12. SPC; Conoxia 100 % Medisinsk Gass, Komprimert; Https://Www.Legemiddelsok.No/_layouts/15/Preparatomtaler/Spc/06-4046.Pdf; Accessed 28.May 2021.

13. Horncastle, E.; Lumb, A.B. Hyperoxia in Anaesthesia and Intensive Care. *BJA Educ* **2019**, *19*, 176–182, doi:10.1016/j.bjae.2019.02.005.

[redacted]

14. Snow, G. *Blockrand: Randomization for Block Random Clinical Trials. R Package Version 1.5. Https://CRAN.R-Project.Org/Package=blockrand*; 2020;

15. Hisdal, J.; Landsverk, S.A.; Hoff, I.E.; Hagen, O.A.; Kirkebøen, K.A.; Høiseth, L.Ø. Associations between Changes in Precerebral Blood Flow and Cerebral Oximetry in the Lower Body Negative Pressure Model of Hypovolemia in Healthy Volunteers. *PLoS One* **2019**, *14*, e0219154, doi:10.1371/journal.pone.0219154.

16. Cecconi, M.; Parsons, A.K.; Rhodes, A. What Is a Fluid Challenge? *Curr Opin Crit Care* **2011**, *17*, 290–295, doi:10.1097/MCC.0b013e32834699cd.
